# The Combination of N-Acetyl Cysteine, Alpha-Lipoic Acid, and Bromelain Shows High Anti-Inflammatory Properties in Novel *In Vivo* and *In Vitro* Models of Endometriosis

**DOI:** 10.1155/2015/918089

**Published:** 2015-04-16

**Authors:** C. Agostinis, S. Zorzet, R. De Leo, G. Zauli, F. De Seta, R. Bulla

**Affiliations:** ^1^Institute for Maternal and Child Health, IRCCS Burlo Garofolo, 34137 Trieste, Italy; ^2^Department of Life Sciences, University of Trieste, Via Valerio 28, 34127 Trieste, Italy; ^3^Department of Medical, Surgical and Health Sciences, University of Trieste, Ospedale di Cattinara, Strada di Fiume 447, 34149 Trieste, Italy

## Abstract

To evaluate the efficacy of an association of N-acetyl cystein, alpha-lipoic acid, and bromelain (NAC/LA/Br) in the treatment of endometriosis we set up a new *in vivo* murine model. We explored the anti-inflammatory and proapoptotic effect of this combination on human endometriotic endothelial cells (EECs) and on endothelial cells isolated from normal uterus (UtMECs). We implanted fragments of human endometriotic cysts intraperitoneally into SCID mice to evaluate the efficacy of NAC/LA/Br treatment. UtMECs and EECs, untreated or treated with NAC/LA/Br, were activated with the proinflammatory stimulus TNF-*α* and their response in terms of VCAM1 expression was evaluated. The proapoptotic effect of higher doses of NAC/LA/Br on UtMECs and EECs was measured with a fluorogenic substrate for activated caspases 3 and 7. The preincubation of EECs with NAC/LA/Br prior to cell stimulation with TNF-*α* prevents the upregulation of the expression of the inflammatory “marker” VCAM1. Furthermore NAC/LA/Br were able to induce EEC, but not UtMEC, apoptosis. Finally, the novel mouse model allowed us to demonstrate that mice treated with NAC/LA/Br presented a lower number of cysts, smaller in size, compared to untreated mice. Our findings suggest that these dietary supplements may have potential therapeutic uses in the treatment of chronic inflammatory diseases like endometriosis.

## 1. Introduction

Endometriosis (EM) is a chronic estrogen-dependent disorder characterized by the presence of endometrium-like tissue outside the uterine cavity. It is associated with dysmenorrhea, dyspareunia, noncyclic pelvic pain, subfertility, and infertility [1]. This frequent gynaecological disease affects 10–15% of women in reproductive age [2]. It is well accepted that a blood supply is essential for the survival of endometriotic implants and the development of EM, as blood is crucial for providing nutrients and growth factors and for promoting recruitment of inflammatory cells to the endometriotic lesions, as described by Groothuis [3]. Endometriotic lesions are highly vascularized, and it is now widely accepted that the formation of new blood vessels at implantation sites plays a key role in the growth of endometriotic cells [4]. Furthermore, eutopic endometrium from women with EM has greater angiogenic potential than eutopic endometrium from healthy subjects [5]. Since the growth of newly formed blood vessels is of pivotal importance in the development of EM, the inhibition of angiogenesis may offer an opportunity for treatment [6–8]. In this respect, it is noteworthy that vascular endothelium is known to play a critical role in regulation of inflammatory processes [9] and that cell adhesion molecules such as vascular cell adhesion molecule-1 (VCAM1), as well as proinflammatory cytokines, play key roles in the pathogenesis of EM [10].

EM can be treated by excising peritoneal implants, deep nodules, and ovarian cysts. Although lesion eradication is considered a fertility-enhancing procedure, the benefit on reproductive performance is moderate [11]. Surgical removal of ectopic lesions represents the first line of intervention for the treatment of EM but is characterized by a relevant percentage of recurrences [12]. In addition, a variety of medical hormonal therapies, that all aimed to reduce the levels of circulating estrogens, are currently available [13]. However, these treatments are often unsatisfactory and cannot be used over long periods of time, due to the occurrence of severe adverse effects [14]. Therefore, new and improved therapeutic solutions that can efficiently reduce lesions with limited side effects and no interference with the patient's fertility are definitely desirable. In this respect, it has been recently shown that N-acetyl cysteine (NAC) effectively treats ovarian endometriosis. In terms of reduction in cysts size, the data reported by Porpora et al. are even more favorable than those granted by the currently adopted hormonal treatments, with the further advantages of fertility preservation and of the virtual absence of undesired side effects [15].

On these bases, the aim of this study was to investigate the effects of an association of NAC, alpha-lipoic acid (LA), and bromelain (Br)* in vivo* after establishing a novel model of EM based on the injection of human endometrial tissue in the peritoneum of SCID mice. In addition, the of NAC/LA/Br combination was analyzed* in vitro* on microvascular endothelial cells isolated from human endometriotic tissues (EECs) as well as in microvascular endothelial cells isolated from human endometrium (UtMECs).

## 2. Materials and Methods

### 2.1. Preparation of the Compound Mixture

All components of the mixture were purchased from Sigma-Aldrich (Milan, Italy) and solutions were sterilized by 0.22 *μ*m filtration. Stock concentrations were 10 mg/mL in H_2_O for NAC, 5 mg/mL in absolute ethanol for LA, and Br 1 mg/mL in PBS for Br. NAC and LA solutions were stored at 4°C while Br at −20°C until use. All reagents were tested for sterility and LPS. For cell culture studies, the three drugs were combined in complete cell culture medium at a final concentration of 1000 *μ*g/mL NAC + 500 *μ*g/mL LA + 50 *μ*g/mL Br. In these conditions, the solution did not form visible precipitates and pH was stable (measured with pH-Meter BASIC20+, CRISON INSTRUMENT). To choose the optimal concentrations for the* in vitro* studies, we have referred to the concentrations of NAC, LA, and Br, proportionally to those present in the new dietary supplement Naxend (Pizeta Pharma, Perugia, Italy; 72.72% NAC, 24.24% LA and 3.03% Br), considering also their bioavailability, the absorption, and the peak plasma of each compound.

### 2.2. Human Tissues

The Maternal-Children's Hospital (RC 08/13, IRCCS “Burlo Garofolo,” Trieste, Italy) approved this study, and following informed consent, endometriosis specimens were obtained from women undergoing laparoscopy to remove endometrial cysts and endometrial tissue was fertile women undergoing hysterectomy for leiomyomatosis in the midproliferative and midsecretory phase defined according to Noyes criteria [16].

### 2.3. Animals

Female SCID mice (4–6 weeks of age) were purchased from Charles River (Milan, Italy) and maintained under pathogen-free conditions. All the experimental procedures involving animals were done in compliance with the guidelines of the European (86/609/EEC) and the Italian (D.L.116/92) laws and were approved by both the Italian Ministry of Health and the Administration of the University Animal House.

### 2.4. Animal Model and* Ex Vivo* Analysis of Cysts

Endometriotic tissue from three peritoneal cysts was collected in sterile PBS and then suspended as coarse fragments, loaded in 3 mL syringes, and standardized with respect to volume and weight. A volume of 0.5 mL of cyst suspension approximately equal to 0.4 g of wet tissue was injected by 16 ga needle intraperitoneally into SCID mice (Charles River). Hormonal therapy with 17-*β*-estradiol-3-benzoate (Sigma-Aldrich, 30 *μ*g/kg i.m.) was initiated at the time of cyst tissue injection and at 3-day intervals thereafter. The day after injection mice were divided randomly in two groups and we administered only to the first one NAC 250 mg/kg/die, LA 125 mg/kg/die, and Br 12,5 mg/kg/die* per os*. Twenty-one days following injection, the animals were killed and implanted endometriotic lesions in treated (*n* = 7) or untreated mice (*n* = 9) were identified, counted, resected, and collected in formalin 10%.

Endometriotic lesions excised from SCID mice were fixed in 10% buffered formalin and paraffin embedded. Four-micrometers-thick sections were stained with Diff-Quick (Biomap, Milan, Italy) staining (following the manufacturer instructions) and examined for the presence and distribution of vessels and glands. For immunohistochemical analysis, the slides were microwaved three times in Tris-HCl/EDTA (ethylenediamine tetraacetic acid) pH 9.0 buffer (Dako) for 5 min, brought to RT, and washed in PBS. After neutralization of the endogenous peroxidase with H_2_O_2_ for 10 min, the sections were first incubated with protein block (Dako) for 10 min and then with the primary antibodies for 1 h at RT (polyclonal rabbit anti-human vWF from Dako). The bound antibodies were revealed using the horseradish peroxidase- (HRP-) conjugated anti-rabbit IgG antibodies (Sigma-Aldrich) and diaminobenzidine (DAB) as substrate (Dako). Slides were evaluated under Leica DM3000 microscope (Leica, Wetzlar, Germany) and the pictures were collected using a Leica DFC320 digital camera (Leica).

### 2.5. Immunofluorescent Staining

Endometriotic tissue fragments approximately 1 cm^3^ were embedded in OCT (BioOptica, Milan, Italy), snap-frozen in liquid nitrogen, and kept at −80°C until use. Cryostat sections of about 6 *μ*m were air dried, fixed in acetone, and either used immediately or kept at −80°C. Binding of mouse anti-human cytokeratin 8/18 (CK8/18) or mouse anti-human vWF (Dako, Milan, Italy) was detected by incubating the sections with goat anti-mouse IgG Cy3-conjugated secondary antibodies 30 min at RT, and then the nuclei were stained blue with DAPI (4′,6-diamidino-2-phenylindole, Sigma-Aldrich) 1 *μ*g/mL.

### 2.6. Cell Isolation and Culture

UtMECs and EECs were isolated and characterized as previously described by Bulla et al. [17]. Both ECs were positively selected with Dynabeads M-450 (Life Technologies, Milan, Italy) coated with* Ulex europaeus* 1 lectin (Sigma-Aldrich), seeded on 12,5 cm^2^ flask precoated with 2 *μ*g/cm^2^ fibronectin (Roche, Milan, Italy), and maintained in serum-free endothelial basal medium (Life Technologies, Monza, Italy) supplemented with 20 ng/mL bFGF (basic Fibroblast Growth Factor), 10 ng/mL EGF (Epidermal Growth Factor), 10% FCS (all from Life Technologies), and 10% human serum and incubated at 37°C, 5% CO_2_. The purity of the resulting EC populations was more than 98% as verified by staining with antibodies to VWF, CD105, VE cadherin (Dako, Milano, Italy), and CD31/PECAM-1 kindly provided by M. R. Zocchi (San Raffaele Hospital, Milan, Italy).

### 2.7. Immunofluorescence on Endothelial Cells

ECs were plated in 8-chamber culture slides (BD Biosciences Discovery Labware, Milan, Italy) coated with 2 *μ*g/cm^2^ fibronectin (Roche) and incubated at 37°C in CO_2_ enriched atmosphere. When cells grew to confluence, they were fixed and permeabilized with FIX & PERM cell permeabilization kit (Società Italiana Chimici, Rome, Italy). Then cells were incubated with primary mAb, (cloneF8/86) mouse anti-human vWF (Dako), or mouse anti-human CD31 (Immunotools, Germany) for 1 h at room temperature (RT) followed by FITC-conjugated goat anti-mouse IgG for 1 h at RT. Images were acquired with Leica DM3000 microscope (Leica) and the pictures were collected using a Leica DFC320 digital camera (Leica).

### 2.8. Cytofluorimetric Analysis

ECs were detached from culture flasks with 5 mM EDTA at 37°C and a total number of 5 × 10^5^ were fixed with FIX & PERM cell permeabilization kit (Società Italiana Chimici) and incubated in permeabilization solution in ice for 30′ with mAb (clone 9) mouse anti-human vimentin (Sigma-Aldrich), mAb (cloneF8/86) mouse anti-human vWF, or mAb (cloneV9) mouse anti-human CK8/18. The binding of primary antibodies was detected by incubation with FITC-conjugated goat anti-mouse IgG. The membrane antigens were detected on unfixed cells, using monoclonal anti-human CD31, CD45, CD34, and CD105 directly FITC-conjugated, all purchased from Immunotools (Germany). The cells were fixed with 1% paraformaldehyde (Sigma-Aldrich) and analyzed for fluorescence with a FACScalibur instrument (BD Falcon, Milan, Italy) using CellQuest software.

### 2.9. Whole Cell VCAM1 ELISA

Both types of cells were grown to the confluence in 96-well plates and incubated with drugs (concentration of NAC 10 *μ*g/mL, LA 9 *μ*g/mL, and Br 2 *μ*g/mL), alone or in association, for 48 h 37°C 5% CO_2_. Successively the cells were stimulated overnight with TNF-*α* (100 ng/mL), washed with Dulbecco's PBS added with 2% BSA (Bovine Serum Albumine, fraction V, Sigma-Aldrich) and CaCl_2_-MgCl_2_ 0,7 mM (Sigma-Aldrich), and then incubated with mouse mAb anti-human VCAM1 (Sigma-Aldrich) 5 *μ*g/mL for 90 min at RT. The binding of primary antibody was reveled incubating the cells with a polyclonal anti-mouse IgG conjugated with alkaline phosphatase. The enzymatic reaction was developed with PNPP (p-nitrophenyl phosphate) (Sigma-Aldrich; 1 mg/mL) as substrate and read kinetically at 405 nm using a Titertek Multiskan ELISA reader (Flow Labs, Milano, Italy).

### 2.10. Apoptosis Assay

Both types of cells were grown to 80% of confluence in 96-well plates and incubated with the compounds, alone or in association, for 72 h 37°C. After then cells were incubated with 5 *μ*M of CellEvent Caspase-3/7 Green Detection Reagent (Life Technologies), a fluorogenic substrate for activated caspases 3 and 7. The reagent consists of a four amino acid peptide (DEVD) conjugated to a nucleic acid binding dye. This cell-permeant substrate is intrinsically nonfluorescent, because the DEVD peptide inhibits the ability of the dye to bind to DNA. After activation of caspase-3 or caspase-7 in apoptotic cells, the DEVD peptide is cleaved, enabling the dye to bind to DNA and produce a bright, fluorogenic response with an absorption/emission maxima of ~502/530 nm. The fluorescence data were acquired with TECAN Infinite200 and normalized for total protein present in each well. For the protein quantitation the cells were then lysed with NaOH 1 M and evaluated by Bradford assay as previously reported [18].

## 3. Statistical Analysis

For each set of experiments, values are reported as means ± SE. The results were evaluated by using the Mann-Whitney test. Statistical significance was defined as *P* < 0.05.

## 4. Results

### 4.1. Establishment of a Relevant* In Vivo* Model for EM and Efficacy of the NAC/LA/Br Combination in Decreasing the Number of Cysts Formation* In Vivo*

**Figure 1:**
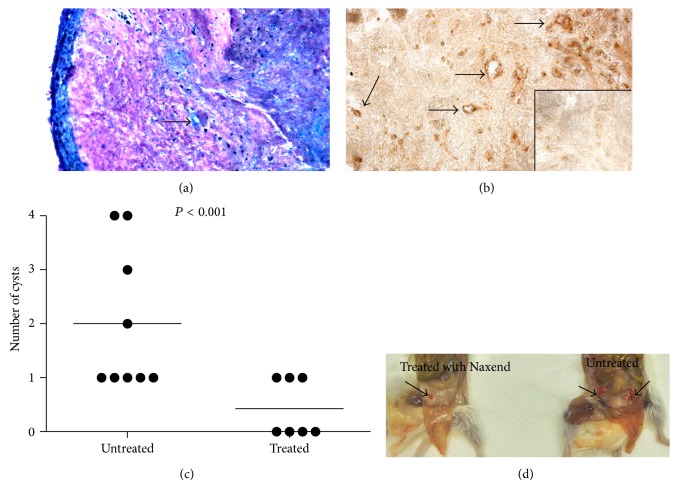
NAC/LA/Br reduces the number and the dimension of cysts in a new* in vivo* model of EM. (a) Histochemical analysis of an endometriotic lesion in mice with Diff-Quick staining. Original Magnification 100x. (b) Immunohistochemical analysis of a cyst excised from the peritoneum of mice. Sections were stained with rabbit anti-vWF followed by a secondary antibody to rabbit IgG HRP conjugated and revealed with DAB. The inset showed the staining obtained with only secondary antibody. Original Magnification 100x. (c) Number of cysts counted in untreated mice (*n* = 9) or treated with NAC 250 mg/kg/die, AL 125 mg/kg/die, and Br 12,5 mg/kg/die (*n* = 7). Mann-Whitney test *P* < 0.001. (d) Representative image of the different morphological appearance of cysts in treated or untreated SCID mice.

The primary aim of our study was the evaluation of the effect of NAC/LA/Br in a relevant model of EM. For this purpose, we set up a new mouse model, modifying the animal model described by Awwad and colleagues [19] and by Grummer and colleagues [20]. Specifically, we used human endometriotic tissue obtained from ovarian cysts instead of normal human endometrium to create the endometriotic lesions into the peritoneal cavity of SCID mice. Endometriotic tissue from three peritoneal cysts was injected in the peritoneal cavity of SCID mice. Hormonal therapy with 17-*β*-estradiol-3-benzoate was initiated at the time of injection and at intervals of 3 days thereafter. Twenty-one days following injection, the animals were killed and implanted endometriotic lesions were identified, analyzed, and characterized. This protocol provided an implantation rate of 100% and the dimension and the histology of these cysts were evaluated. Excised explants revealed the presence of EM-like features upon histologic examination, that is, stroma and endometrial glands. All the implants showed a well restructured columnar and/or cuboidal glandular epithelium with cytogenetic stroma. A nascent capillary network was present at the interface between the implant and the underlying murine tissue. The presence of new vessels, indicated with black arrows in Figure 1(a), was confirmed by immunohistochemistry, staining the sections with anti-vWF polyclonal antibodies (Figure 1(b)). For these* in vivo* experiments, we treated the animals daily with NAC (250 mg/kg), LA (125 mg/kg), and Br (12,5 mg/kg) provided in the animals' water bottles, a* per os* administration that mimics human dosing [21]. To choose the optimal concentrations of NAC, LA, and Br, we have referred to the concentrations of NAC, LA, and Br, proportionally to those present in the new dietary supplement Naxend (Pizeta Pharma, Perugia, Italy; 72.72% NAC, 24.24% LA, and 3.03% Br).

As shown in Figure 1(c), all control group animals developed at least 1 cyst, with up to 4 in some control animals. In 4 mice treated with the NAC/LA/Br combination, no cyst was visible and in 3 mice only 1 cyst was present. The number of cysts developing in treated compared to untreated animals was significantly (*P* < 0.05) lower (Figure 1(d)). It is also noteworthy that the cysts present in the untreated animal were larger than those in the treated mice.

### 4.2. Isolation and Characterization of Endometriotic Endothelial Cells (EECs) and Evaluation of Their* In Vitro* Response to the NAC/LA/Br Combination

**Figure 2:**
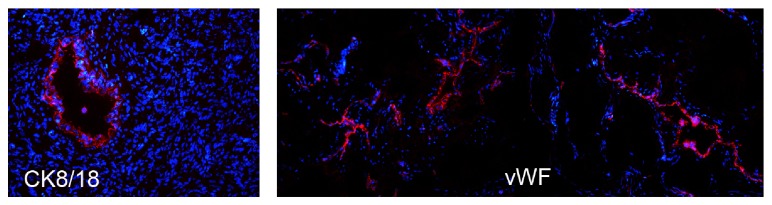
Immunofluorescence analysis of human endometriotic cysts. The frozen sections were stained with mAb anti-human vWF to highlight the vessels or with mAb anti-human CK 8/18 to show the glands. The binding of mouse monoclonal antibodies was revealed by the incubation with goat anti-mouse IgG Cy3-conjugated secondary antibodies. Nuclei were stained in blue by DAPI: original magnification 100x.

**Figure 3:**
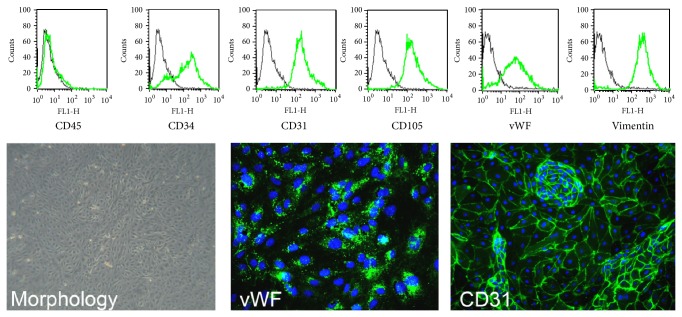
Characterization of the purity of endothelial cells isolated from endometriotic tissue. (A) EECs were characterized by cytofluorimetric analysis for the expression of CD45, CD34, CD31, CD105, vWF, and vimentin, and the expression of these markers (green lines) was compared with correlated control antibodies (black lines). The expression of vWF and CD31 was confirmed by immunofluorescence, on EECs grown to confluence in 8-chamber culture slides. After fixation and permeabilization the cells were stained with mAb anti-human CD31 or anti-vWF. The binding of mouse monoclonal antibodies was revealed by the incubation with goat anti-mouse IgG FITC-conjugated secondary antibodies. Nuclei were stained in blue by DAPI: original magnification 200x.

Since endothelial cells play a significant role in the development of endometriotic lesions [4], as also confirmed in our animal model, we next developed a new protocol for the isolation and culture of endothelial cells from human endometriotic ovarian cysts. For this purpose, the presence and the density of the vessels inside human endometriotic cysts were initially analyzed by immunofluorescence on sections of human endometriotic tissues. The sections were stained with mAb anti-human vWF, a classical endothelial cell marker, and with CK8/18, in order to evidence the presence of endometriotic glands.  Figure 2 clearly shows the presence of several vessels inside the cysts and the presence of one representative gland. These samples were then used to isolate the endothelial cells. The isolated endothelial cells cultured on fibronectin easily reach confluence within few days. The morphology of cultured EECs stained with mAb anti-CD31, another typical marker of endothelial cells, is shown in Figure 3. We then characterized EECs by cytofluorimetric analysis in order to evaluate the purity of these cells and to exclude the presence of contaminating cells. 100% of these cells were positive for classical endothelial cell markers (CD31, CD105, vWF, and vimentin) and 80% were positive for CD34, a marker for newly formed vessels (Figure 3). Cultured EECs were devoid of expression of the epithelial marker CK8/18 and leukocyte marker CD45. Consistent results were obtained for EEC populations derived from five different patients.

### 4.3. NAC/LA/Br Exerts Anti-Inflammatory Effect Reducing the Expression of VCAM1 in TNF-*α* Stimulated EECs

**Figure 4:**
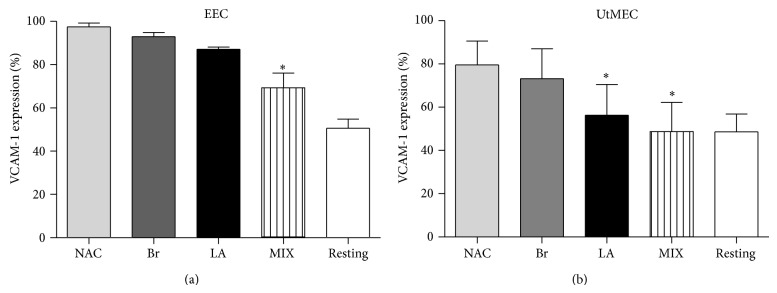
Anti-inflammatory effect of NAC/LA/Br on ECs: analysis of VCAM1 expression. Five different populations of endometriotic endothelial cells (EECs) and 5 different populations of uterine microvascular endothelial cell (UtMECs) were isolated, as described by Bulla et al. [17] with some modifications. ECs were grown to confluence in a 96-well plate and then incubated with NAC 10 *μ*g/mL, AL 9 *μ*g/mL, and Br 2 *μ*g/mL, alone or in association (MIX). Successively the cells were stimulated overnight with TNF-*α* (100 ng/mL) and incubated with anti-human VCAM1. The binding of primary antibody was revealed incubating the cells with a goat anti-mouse IgG conjugated with alkaline phosphatase. The 100% of VCAM1 expression is referred to the TNF-*α*-treated cells. Data are expressed as mean ± SE of results from five experiments each performed in triplicate. ^*^ *P* < 0.05 with respect to the untreated (Mann-Whitney test).

In next group of experiments, EECs from 5 distinct patients were grown to confluence and then stimulated with 100 ng/mL of the proinflammatory cytokine TNF-*α* in order to upregulate the expression of the adhesion molecule VCAM1 [22], which represent a proinflammatory marker. We compared the total amount of VCAM1 by ELISA on EECs untreated, EECs stimulated for 12 hours with TNF-*α*, and cells treated with TNF-*α* previously preincubated for 72 hours with NAC, LA, and Br, (NAC 10 *μ*g/mL, AL 9 *μ*g/mL, and Br 2 *μ*g/mL) used alone or in association. No reduction in VCAM1 expression was observed in cells treated with individual drugs (Figure 4(a)). Only the drug combination exerted a statistically (*P* < 0.05) significant although incomplete decrease of VCAM1 levels as compared to TNF-*α*-treated cultures. For comparison, we have used endothelial cells isolated from normal human endometrium from women undergoing hysterectomy (UtMECs) [17]. As shown in Figure 4(b), the downregulation of VCAM1 in TNF-*α*-treated UtMECs was complete in the presence of the combination of NAC + Br + LA (MIX). The addition of NAC/LA/Br to the endothelial cell culture media did not alter the medium pH and no effect to the cell viability was observed for ECs treated with the same concentrations of compounds as assessed by trypan blue staining (data not shown).

### 4.4. NAC/LA/Br Combination Selectively Exerts Proapoptotic Activity on EECs

**Figure 5:**
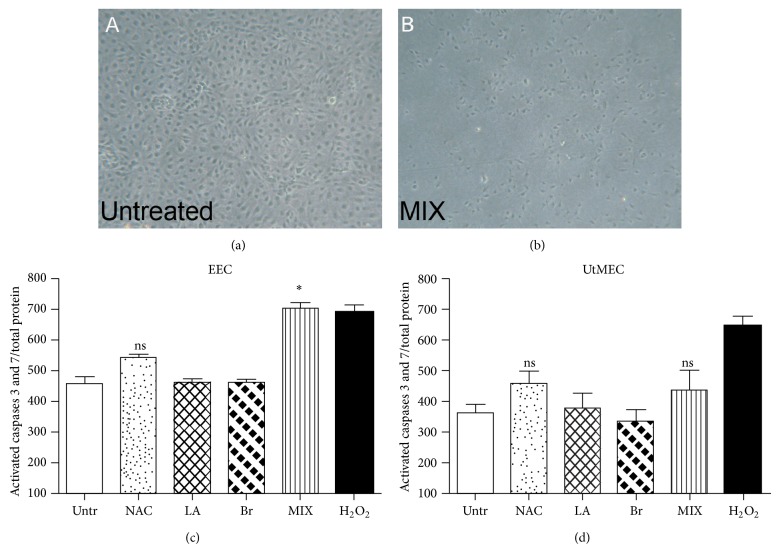
Evaluation of the proapoptotic effect of NAC/LA/Br on ECs. (a), (b) Morphologic appearance of EECs untreated (a) or incubated with NAC 20 *μ*g/mL, AL 18 *μ*g/mL, and Br 4 *μ*g/mL in association (MIX) (b). (c), (d) Both types of cells were grown to 80% of confluence in 96-well plates and incubated with NAC 20 *μ*g/mL, AL 18 *μ*g/mL, and Br 4 *μ*g/mL, alone or in association (MIX), for 72 h 37°C. The cells were then incubated with 5 *μ*M of CellEvent Caspase-3/7 Green Detection Reagent (Life Technologies), a fluorogenic substrate for activated caspases 3 and 7. The fluorescence data were normalized for the total protein present in each well. Data are expressed as mean ± SE of results from three experiments each performed in triplicate. ^*^ *P* < 0.05 with respect to the untreated (Mann-Whitney test).

In order to evaluate whether besides the anti-inflammatory activity the NAC, LA, and Br mixture might also affect endothelial cell viability, EECs were plated on 8-chamber culture slides, grown to 80% confluence, and incubated for 72 h with NAC, LA, and Br (NAC 20 *μ*g/mL, AL 18 *μ*g/mL, and Br 4 *μ*g/mL) used alone or in combination. As shown in Figures 5(a)-5(b), EECs incubated with the mixture of compounds showed a decreased number of viable cells as compared to untreated cultures. Based on these results, we then investigated the ability of NAC/LA/Br to induce apoptosis. Both EECs and UtMECs were incubated with a fluorogenic substrate for activated caspases 3 and 7 and the fluorescence values obtained were normalized for total protein present in each well. As shown in Figure 5(c), treatment of the cells with the NAC/LA/Br mixture, at these higher concentrations, was able to induce a statistically significant (*P* < 0.05) increase of apoptosis of EECs. In fact, the induction of caspase activity induced by the drug combination was comparable to that of positive control (H_2_O_2_). Of note, the NAC/LA/Br mixture was totally ineffective in UtMECs (Figure 5(d)).

## 5. Discussion

Most recent guidelines for the treatment of endometriosis-associated symptoms recommend to surgically treat endometriosis, as this is effective for reducing endometriosis-associated pain for those in whom medical treatment has failed [23]. Medical treatments for EM are usually aimed at reducing the production of endogenous estrogens or inducing endometrial differentiation with progestins. The pain associated with endometriosis is usually treated initially with oral contraceptive agents or non-steroidal anti-inflammatory drugs, because these agents have fewer side effects and are less expensive than other treatment options. GnRH agonists, as well as other agents such as danazol or progestational agents, and, recently, aromatase inhibitors are usually reserved for use if the first-line agents fail to provide an acceptable degree of relief. These agents represent standard therapies for EM but are associated with long-term side effects [14]. Although currently available medical therapies are not curative* per se*, they are important for pain suppression and lesion regression. Thus, efforts are still being focused on the improvement and promotion of new treatments with higher efficacy and fewer side effects. A high number of medications have been tested in preclinical models of endometriosis due to their theoretical capacity of disrupting important pathophysiologic pathways of the disease, such as inflammatory response, angiogenesis and cell survival, proliferation, migration, adhesion, and invasion. TNF-*α* blockers, nuclear factor kB inhibitors, antiangiogenic agents, statins, antioxidants, immune-modulators, flavonoids, histone deacetylase inhibitors, matrix metalloproteinase inhibitors, metformin, novel modulators of sex steroids expression, and apoptotic agents were all effective* in vitro* and/or in animal models. Most of these agents did not reach the clinical setting, mainly because of the high risk of adverse effects [24].

An alternative approach for treatment of EM is represented by anti-inflammatory compounds [11]. In particular, Pittaluga and colleagues [25] and Onalan et al. [26] recently demonstrated the efficacy of NAC in two different* in vivo* models of EM. No data are available on the use of LA and Br for the treatment of EM but they are currently in clinical use for the treatment of inflammatory diseases [27, 28]. Endometriotic lesions are highly vascularized, and it is now widely accepted that the formation of new blood vessels in implanted places plays a key role in the growth of endometriotic cells [4]. The growth of newly formed blood vessels is of pivotal importance in the development of EM, so inhibition of angiogenesis may offer a new opportunity for treatment [29]. Therefore, in this study we have proposed an innovative* in vitro* model to study the efficacy of a new treatment for EM, based on the analysis of endometriotic endothelial cell response, in consideration of the key role of endothelial cells in controlling inflammation and angiogenesis. A first important achievement of our study was the establishment of a novel mouse model based on the intraperitoneal injection of endometriotic human tissue in SCID mice. In analogous models available in the literature, fragments of human endometriotic cysts were surgically fixed to the peritoneal wall [20]. The benefit of our model is that our procedures reduce animal suffering and animal losses. The fact that all treated animals developed at least one cyst is a profoundly beneficial aspect of this model. Immunohistochemical observations indicated that the tissue excised from murine peritoneum developed several new vessels, indicating that the cysts had a morphological organization similar to human cysts with new blood vessel formations. Thanks to the development of this mouse model we have been able to demonstrate the effectiveness of NAC/LA/Br* in vivo* with the result that treated mice presented a lower number of cysts, which were also smaller in size than those in untreated mice.

A second important finding of our study was the successful isolation of pure endothelial cells from human endometriotic lesions. Using these cells to set up an* in vitro* model that exploits the endothelial cells is preferable, since many differences exist between endothelial cells isolated from different sites [30]. Our results indicated that when used alone the three studied compounds are able to induce only a modest or null inhibition of TNF-*α* activation of VCAM1 and a strong inhibition when used in combination, suggesting the presence of an additive effect of the three compounds. In line with our current data previous findings obtained on hypertensive patients (with type 2 diabetes) treated with NAC experienced reduction of C-reactive protein, intracellular adhesion molecule, and vascular cell adhesion molecule [31]. In addition, Tisato et al. documented that LA significantly decreased the baseline levels of PDGF, RANTES, and CXCL10 expression and counteracted TNF-*α*-induced NF-B and p38/MAPK activation in endothelial cells from chronic venous disease patients [27]. A second important finding of our study was the ability of NAC/LA/Br to promote apoptosis in EEC. In this respect, it should be underlined that the* in vitro* behavior of UtMECs was different as these cells were totally unaffected by the NAC/LA/Br combination in terms of apoptosis induction. These findings underline the importance not only of tissue-specificity but also of pathological specificity of endothelial cells. This might explain the partial discrepancies of our current data with those of Cai et al. [32] and Mohr and Desser [33] which, respectively, indicated that Br inhibits endothelial cell invasion and angiogenesis, while Larghero et al. [34] demonstrated that LA induced apoptosis through the production of the proapoptotic TNF-alpha-related apoptosis-inducing ligand (TRAIL) cytokine in endothelial cells [34].

## 6. Conclusions

In conclusion, we have adopted an improved* in vivo* model of EM, which reduces animal suffering by nonsurgical implantation of tissue from human cysts. Moreover, EECs are a unique, human-derived, easily available,* in vitro* model of EM that may advance study of the inflammatory process and the role of angiogenesis in endometriosis. Thanks to these models, we could demonstrate that NAC/LA/Br is an effective treatment for EM that may have potential therapeutic uses in the prevention and treatment of patients. It would be interesting in a further study to compare the effect of NAC/LA/Br with standard therapies and to evaluate if the use of NAC/LA/Br in combination with standard therapies may lead to the improvement of the standard medical treatment for EM.
